# Premarin Reduces Neurodegeneration and Promotes Improvement of Function in an Animal Model of Spinal Cord Injury

**DOI:** 10.3390/ijms23042384

**Published:** 2022-02-21

**Authors:** Azizul Haque, Arabinda Das, Supriti Samantaray, Denise Matzelle, Mollie Capone, Gerald Wallace, Aarti N. Husarik, Saied Taheri, Russel J. Reiter, Abhay Varma, Swapan K. Ray, Naren L. Banik

**Affiliations:** 1 Department of Microbiology and Immunology, Medical University of South Carolina, Charleston, SC 29425, USA; bouchard@musc.edu; 2 Department of Neurosurgery, Medical University of South Carolina, Charleston, SC 29425, USA; dasa@musc.edu (A.D.); samantasupritiray@gmail.com (S.S.); matzeldd@musc.edu (D.M.); gerald_wallace4@hotmail.com (G.W.); aartihusarik@gmail.com (A.N.H.); varma@musc.edu (A.V.); 3 Ralph H. Johnson VA Medical Center, Charleston, SC 29401, USA; 4 Department of Pharmaceutical Sciences, College of Pharmacy, University of South Florida, Tampa, FL 33620, USA; taheris@health.usf.edu; 5 UT Health Science Center at Houston, Department of Cellular and Structural Biology, University of Texas, San Antonio, TX 78229, USA; reiter@uthscsa.edu; 6 Department of Pathology, Microbiology, and Immunology, School of Medicine, University of South Carolina, Columbia, SC 29209, USA; swapan.ray@uscmed.sc.edu

**Keywords:** spinal cord injury, inflammation, Premarin, estrogen, neuroprotection

## Abstract

Spinal cord injury (SCI) causes significant mortality and morbidity. Currently, no FDA-approved pharmacotherapy is available for treating SCI. Previously, low doses of estrogen (17β-estradiol, E2) were shown to improve the post-injury outcome in a rat SCI model. However, the range of associated side effects makes advocating its therapeutic use difficult. Therefore, this study aimed at investigating the therapeutic efficacy of Premarin (PRM) in SCI. PRM is an FDA-approved E2 (10%) formulation, which is used for hormone replacement therapy with minimal risk of serious side effects. The effects of PRM on SCI were examined by magnetic resonance imaging, immunofluorescent staining, and western blot analysis in a rat model. SCI animals treated with vehicle alone, PRM, E2 receptor antagonist (ICI), or PRM + ICI were graded in a blinded way for locomotor function by using the Basso–Beattie–Bresnahan (BBB) locomotor scale. PRM treatment for 7 days decreased post-SCI lesion volume and attenuated neuronal cell death, inflammation, and axonal damage. PRM also altered the balance of pro- and anti-apoptotic proteins in favor of cell survival and improved angiogenesis and microvascular growth. Increased expression of estrogen receptors (ERs) ERα and ERβ following PRM treatment and their inhibition by ER inhibitor indicated that the neuroprotection associated with PRM treatment might be E2-receptor mediated. The attenuation of glial activation with decreased inflammation and cell death, and increased angiogenesis by PRM led to improved functional outcome as determined by the BBB locomotor scale. These results suggest that PRM treatment has significant therapeutic implications for the improvement of post-SCI outcome.

## 1. Introduction

Spinal cord injury (SCI) is a devastating problem affecting injured individuals and leading to disability. Depending on the extent and severity of injury, SCI can lead to varying degrees of paralysis. Unfortunately, no Federal Drug Administration (FDA) approved pharmacotherapies are available to cure and/or improve motor function following injury. While methylprednisolone is a widely used drug for SCI treatment, its use is controversial and has many unwanted side effects as well as weak efficacy [1,2,3,4,5]. The multi-factorial destructive process in SCI makes developing pharmacologic agent(s) that can ameliorate the many destructive pathways involved in spinal cord degeneration difficult.

17β-Estradiol (E2), a naturally occurring hormone, has been found to attenuate many of the destructive processes and to provide beneficial effects following SCI [6,7]. Administration of both E2 and melatonin (also a naturally occurring hormone) has been found to improve locomotor function in SCI by modulating Ca^2+^ influx and many other common factors involved in tissue degeneration and cell death. Both neurohormones have been shown to promote neuroprotection by attenuating increased activities of free radicals, calpain (calcium-activated protease), and other detrimental factors involved in SCI. While the majority of SCI studies have found E2 treatment to be beneficial, standardization of E2 dose and time of administration is of utmost importance for clinical relevance [8,9]. Previous studies from different laboratories, including our own, have used very high non-physiological doses of E2, ranging from 50 μg to 4 mg/kg, for treatment of rats following SCI [7,10,11,12,13]. The results from these studies indicated attenuation of inflammation, protection of cells, tissue sparing, and improvement of function.

These early findings provided solid evidence for the potential use of E2 as a therapeutic agent for SCI. Our recent studies have indicated administration of low dose E2 (10 μg) in rats following SCI protected cells, axons, and myelin in acute injury and improved locomotor function following chronic injury in rats [14,15,16]. Similarly, use of melatonin by many investigators has shown improvement of locomotor function in spinal cord injury [17,18,19]. Importantly, the cytoprotective effects of E2 and melatonin in combating both oxidative stress and glutamate toxicity, both of which are prominent players in neurotrauma have been demonstrated in vitro [20,21,22,23]. These effects are mediated by E2 through E2 receptors (ERs), which reduce microglial activation, NF-κB, and iNOS [24]. However, E2 therapies can increase risk for embolism and cancer as well as promote feminizing effects in males, which limits the use of E2 in clinical trials [25,26,27,28]. Instead, Premarin^®^ (PRM), a conjugated form of low dose E2 that is FDA-approved, is associated with minimal risk of serious side effects and is currently used in E2 replacement therapy [29,30].

PRM is composed of 8–10% E2, but it also contains progestin, estriol, and estrones [31]. While the composition of PRM is complex, the presence of E2 in PRM has been found to provide neuroprotection after traumatic brain injury (TBI) [32]. Although PRM has been found to have beneficial effects in TBI, its neuroprotective effect at a high dose (1 mg of PRM ≈ 100 μg of E2) was demonstrated in SCI in rats [33]. Since unwanted side effects from such a high dose will prevent use of PRM in clinical settings, the effect of low doses PRM (10–100 μg PRM ≈ 1–10 μg E2) was examined in rats. The results from these experiments demonstrate that PRM efficacy is not due to progestin or other sterols as the ER antagonist ICI182780 blocks ER-mediated beneficial effects by upregulating inflammatory factors (COX-2, NF-κB) and death genes. In contrast, E2 and ER receptor agonists has been found to protect neurons from the toxic effects of TNFα-induced apoptosis [34]. However, PRM treatment also protected neurons, attenuated axonal degeneration, and promoted blood vessel growth in SCI compared to untreated control animals. The results obtained from PRM studies are very similar to those from E2 treatment and were presented at the American Society for Neurochemistry meeting in an abstract form [35]. Therefore, these findings with low dose PRM (100 μg PRM = 10 μg E2) represent establishment of a safe conjugated E2-based therapy that can be swiftly transitioned from bench to bedside.

## 2. Materials and Methods

### 2.1. Induction of SCI and Treatment with PRM

**Figure 1:**
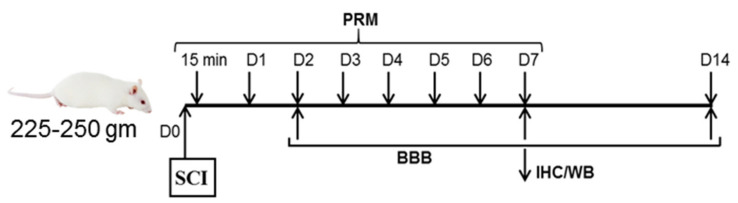
The flow chart indicates experimental overview. A 1 cm section of spinal cord centered on the lesion as well as 1 cm rostral (starting 0.5 cm rostral from the impact epicenter) and 1 cm caudal (starting 0.5 cm caudal from the impact epicenter) were collected for all groups so that the location and size remained the same for quantitative analysis. This study tested longitudinal lesion sections (1/2 + 1/2) by both immunohistochemistry and western blotting. All analyses were performed in a blinded fashion using standardized protocols [14,16,38].

Male Sprague–Dawley (Envigo, Dublin, VA, USA) rats weighing 225–250 mg were used for these studies. ARRIVE guidelines were followed and all the animal experiments were conducted in accordance with the guidelines of Institutional Animal Care and Use Committee (IACUC) at the Medical University of South Carolina (MUSC), Charleston, SC (protocol ARC no. 2079). All animal studies were approved by the IACUC at the MUSC. Animals were anesthetized with ketamine (Ketaset at 80 mg/kg, Pfizer, New York, NY, USA) and xylazine (AnaSed at 10 mg/kg, Lloyd laboratories, Shenandoah, IA, USA) as described [14]. Laminectomies were performed at T10 and the spine immobilized with a stereotactic device, and SCI was induced by the method of Perot by dropping a 5 g weight from a height of 8 cm onto an impounder tip gently placed on the exposed spinal cord [36,37]. Sham animals receiving laminectomy but no injury were used as controls (*n* = 4–10). PRM (100 µg/kg), E2 (10 µg/kg), and/or ICI (1 mg/kg) were given a bolus dose via tail vein injection at 15 min post-injury followed by a daily intraperitoneal injection up to day 7 (*n* = 4–10) (Figure 1). Animals were sacrificed by decapitation under ketamine/xylazine anesthesia at 7 days following injury.

### 2.2. Magnetic Resonance Imaging (MRI)

MRI was performed using a Bruker 7T/300 MHz spectrometer (Billerica, MA, USA) equipped with a 12 cm high performance BGA-S gradient and shim coil set, capable of generating a maximal gradient amplitude of 660 mT/m with a rise time of 80–120 µs. Animals from control or PRM-treated groups were anesthetized using gaseous isoflurane in oxygen (3% box induction) and maintained at 0.5–2.5% during all MRI procedures. An MRI-compatible monitoring and gating system (Model 1025, SA Instruments Inc., Stony Brook, NY, USA) was used to monitor heart rate, respiration rate, and body temperature during scanning to monitor the wellbeing of animal and to perform physiologically gated image acquisition to further reduce motion artifact. Warm air was circulated in the magnet bore to ensure a stable 37 °C body temperature throughout the imaging session. After the hindlimb was shaved and cleaned with alcohol, a 1 × 1 cm circular ^31^P (190.5 MHz) tuned surface coil was placed over the belly of the gastrocnemius muscles. A 3-cm standard ^1^H surface coil was placed underneath the hind limb to perform shimming and the hind limb was extended such that the calf muscles were centered over the surface coil. An inflatable blood pressure cuff connected to a source of air outlet on the wall was positioned around the animal’s thigh. Adequate care was taken to stabilize both the coil and the limb to avoid any movement during cuff inflation. Vital signs were monitored throughout the experimental procedure.

### 2.3. Immunofluorescent Staining

Immunofluorescent identification of markers for apoptotic cell death, angiogenesis, inflammation, and neuronal and vascular cells were performed on rat spinal cord samples embedded in Tissue-Tek OCT freezing media, as described previously [14,15]. Longitudinal spinal cord lesion sections (10 µm) were obtained using a Leica CM1850 cryostat (Leica, Deerfield, IL, USA), at −20 °C, air-dried, fixed in 95% ethanol, rinsed in phosphate-buffered saline (PBS, containing 137 mM NaCl, 2.7 mM KCl, 11.9 mM phosphates, pH 7.4), and stored in the same buffer at 4 °C for further studies. After blocking with 2% horse serum in PBS, sections were incubated with primary IgG antibodies.

Immunofluorescence studies included identification of cells with specific marker antibodies, including neurofilament protein (NFP, 1:200) (Catalog: N4142, Sigma-Aldrich, Saint Louis, MO, USA), myelin basic protein (MBP, 1:250) (Catalog: MAB384, Millipore, Burlington, MA, USA), cyclooxygenase-2 (COX-2), Glial fibrillary acidic protein (GFAP, 1:400) for astrocytes (Chemicon, Hampton, NH, USA), dephosphorylated neurofilament (deNFP, 1:500) (SMI311, Sternberger International) for axonal damage, COX-2 (1:200, Catalog: 12282, cell signaling), as well as CD31 (1:100, Chemicon, Hampton, NH, USA), GLUT1 (1:500, Chemicon, Hampton, NH, USA), vascular endothelial growth factor (VEGF, 1:400), and Tie-2 (1:200, Catalog: SC9026, Santa Cruz Biotechnology, Dallas, TX, USA). Following removal of primary antibodies, all spinal cord sections were washed with PBS. Sections were then incubated with IgG secondary antibodies, which were with the desired fluoresce markers.

Double immunofluorescence staining was performed for co-localization of cell death in neurons. First, Terminal deoxynucleotidyl transferase recombinant–mediated dUTP nick end labeling (TUNEL) was performed following the manufacturer’s protocol (Promega, Madison, WI, USA). Spinal cord sections were prefixed in 95% ethanol, immersed in 4% methanol-free formaldehyde, sufficiently washed in PBS, equilibrated in TUNEL reaction buffer, incubated with digoxigenin labeled nucleotides (Roche, Indianapolis, IN, USA), and recombinant TdT enzyme (Promega, Madison, WI, USA) using a humidified Hybaid OmniSlide Thermal Cycler (Hybaid, Ltd., Teddington, UK) to detect TUNEL positive cells with red fluorescence. TUNEL reaction in the spinal cord sections was terminated with 2× NaCl/Na-citrate solution, and unincorporated nucleotides were removed by washing three times in PBS. After completion of the TUNEL reaction, spinal cord sections were stained overnight at 4 °C with the neuronal nuclei marker NeuN (1:100) primary antibody for green fluorescence.

All antibodies used in this study were tested and validated in the SCI tissues from rats in our laboratories for producing results for publications [7,15,18,38]. All fluorescence images were analyzed by using ImageJ software of the National Institutes of Health (NIH), Bethesda, MA, USA) to determine fluorescent pixel intensity at a region of interest (ROI) and report as arbitrary units [39].

### 2.4. Luxol Fast Blue (LFB) Staining

For LFB staining, lesion SCI samples (longitudinal section) were fixed overnight in a 4% paraformaldehyde solution in PBS. After fixation, samples were embedded in paraffin and then sliced sections (10 µm) were mounted on slides. LFB staining was carried out as reported previously [7]. Briefly, slides were stained overnight in 0.1% LFB (Solvent Blue 38; Sigma-Aldrich, Saint Louis, MO, USA) in acidified 95% ethanol. Stained sections were viewed on an Olympus microscope (BH-2; Olympus, Melville, NY, USA) at 20× magnification and captured with a Magna Fire SP CCD camera (Optronics, Goleta, CA, USA). Image capture was done using Magna Fire SP 2.1 software (Optronics, Nuremberg, Germany). SCI tissue was stained so that myelin appeared blue.

### 2.5. Western Blot Analysis

Approximately 50 to 100 μg of tissue was used for Western blot analysis. Spinal cords were homogenized in homogenizing buffer (50 mM Tris-HCl, pH 7.4; 5 mM EGTA; 1 mM phenylmethylsulfonyl fluoride) on ice for protein lysates. The concentration of protein was determined by Coomassie Plus^TM^ Protein Assay Reagent (Pierce, Rockford, IL, USA) read at 595 nm. Samples were mixed with sample buffer (62.5 mM Tris-HCl, pH 6.8, 2% sodium dodecyl sulfate, 5 mM β-mercaptoethanol, 10% glycerol) at 1:1 *v*/*v*, boiled, and briefly spun to collect supernatant. Samples were diluted to a final protein concentration of 1.5 mg/mL with a mixture of homogenizing and sample buffers (1:1 *v*/*v*) containing bromophenol blue (0.01%), reduced and boiled, and loaded onto pre-cast gradient gel (4–20%; Bio-Rad Laboratories, Hercules, CA, USA). Gels were run at 100 V for 60 min, and proteins were transferred to Immobilon^TM^-P polyvinylidene fluoride microporous membranes (Millipore, Bedford, MA, USA) [15,18]. The amount of protein was calculated as percent compared to sham vehicle. Primary antibodies included Bax (B-9, sc-7480), pAkt (Thr-308, #72956, Cell signaling), MBP, NF-κB, COX-2, and p-BCL-2 (sc-293129, 662-ser70) as markers for inflammation and cell death; ERα, ERβ, and GPR30 for E2 receptor levels; Ang-1, (Santa Cruz Biotechnology, Dallas, TX, USA), Flt-1, and Flk-1 at 1:250 dilution to identify angiogenic markers. β-actin (ACT BD11B7, Ssc-81178) was used as a loading control. Secondary antibodies used were goat anti-mouse IgG- horseradish peroxidase (HRP) (SC-2005, Santa Cruz Biotechnology, Dallas, TX, USA), and goat anti-rabbit IgG-HRP (SC-2004, Santa Cruz Biotechnology, Dallas, TX, USA). The optical density (OD) of the protein immunoreactive bands on the Western blot were analyzed with the use of NIH ImageJ software.

### 2.6. Assessment of Locomotor Function (BBB Scoring)

SCI animals treated with vehicle (saline), ICI, PRM, or PRM+ICI were graded weekly by trained personnel using the Basso–Beattie–Bresnahan (BBB) scale as previously reported [14,40,41]. These outcome measurements were supplemented by histochemical and biochemical endpoints as described [14].

### 2.7. Statistical Analyses of Data

Statistical analyses were performed using StatView software (Abacus Concepts, Piscataway, NJ, USA) and GraphPad Prism 8 software (San Diego, CA, USA). Western blot densitometric data is expressed as mean +/− standard error of the mean (SEM) of separate experiments and compared by one-way analysis of variance (ANOVA) followed by Fisher’s post-hoc test. Differences between each group is expressed as *p* < 0.05. Two-way ANOVA was used for BBB. For the quantitation of immunohistochemistry and a part of the Western blot data, the Student’s *t*-test was also used. In all experiments, vehicle-treated rats were used as controls for comparing the other groups. Statistical analyses were performed in a blinded fashion.

## 3. Results

### 3.1. PRM Decreases Post-SCI Lesion Volume

**Figure 2:**
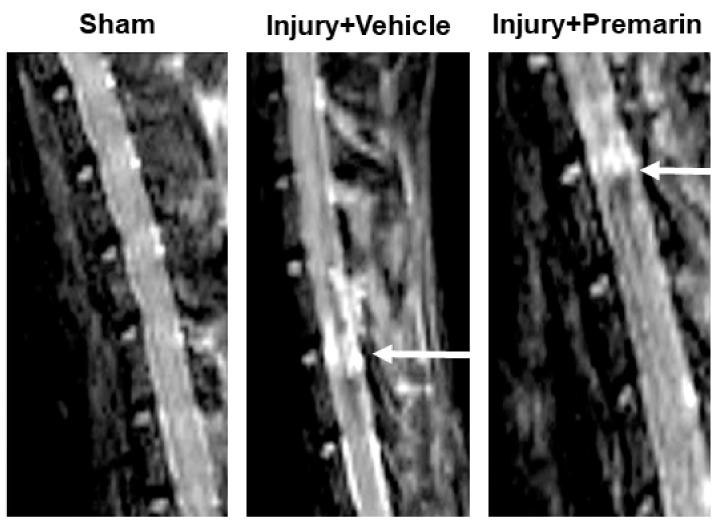
PRM reduces edema and preserves tissue integrity at day 7 following SCI. MRI imaging of spinal cords from sham and injury animals treated with either vehicle or PRM were imaged using a 7 Tesla (T) Bruker BioSpec USR system (Billerica, MA, USA). Imaging in the injured cords treated with vehicle clearly showed the injury lesion when compared to sham animals. PRM treatment decreased edema and preserved tissue integrity at injury site shown by a reduced lesion volume compared to that of the vehicle-treated injured rats. Lesion site is indicated by white arrow.

MRI imaging detected significant lesions at the injury site in vehicle-treated SCI rats in comparison to the sham animals. PRM treatment significantly reduced the lesion volume at 7 days following SCI (Figure 2), which indicates a role for PRM in reducing edema in the injured spinal cord and preserving tissue integrity following SCI.

### 3.2. PRM Attenuates Neuronal Cell Death and Axonal Damage after SCI

**Figure 3:**
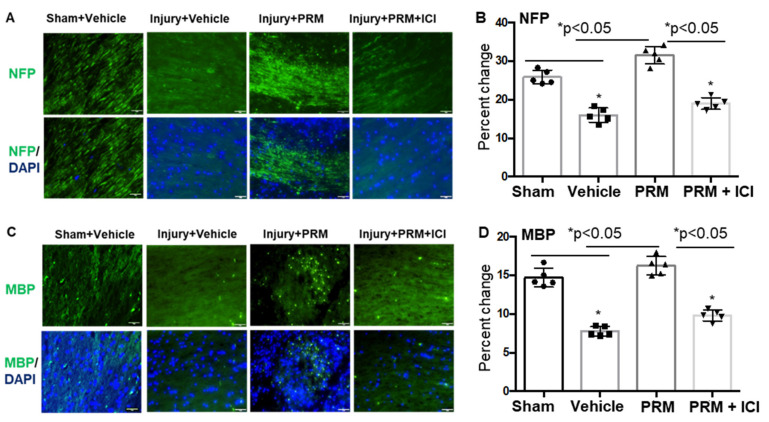
PRM treatment increased neurogenesis and decreased myelin damage at day 7 following SCI. Increased levels of immunostaining of NFP (**A**,**B**) and MBP (**C**,**D**) were detected in longitudinal lesioned sections of injured rats following treatment with PRM. These NFP and MBP levels were significantly improved in PRM treated rats when compared with vehicle treatment. In contrast, there was a significant reduction of NFP protein expression in PRM + ICI treated rats as compared to PRM, indicating the effects of PRM treatment. Similarly, MBP expression levels were also markedly reduced in PRM + ICI treated rats as compared to PRM. These data suggest that neuronal growth as well as myelination are improved following treatment of SCI rats with PRM. Representative immunofluorescent staining images were shown (Magnification, 20×, *n* = 5–6). Statistical analysis was performed by one-way ANOVA followed by Fisher’s post-hoc test. Error bars are standard deviations. (* *p* < 0.05).

Immunostaining of SCI samples showed an increase in the number of NFP and MBP stained cells in injured rats following treatment with PRM (Figure 3). In contrast, a significant reduction of NFP and MBP expression was seen in vehicle-treated injured rats as well as rats treated with a combination of both PRM and ICI inhibitor (Figure 3A–D). Overall, MBP+ cells are better protected by PRM treatment as compared to injury + vehicle, which is evident as demonstrated by the lightly stained damaged cells in the Inj + Veh panel. Again, PRM provided more protection even to the damaged cells in the Inj + PRM while that protection was reduced in the presence of ICI. These data suggest that PRM treatment may preserve neurofilaments and support axonal protection with a reduction of myelin damage following SCI.

The extent of loss of myelin was also analyzed in the lesioned section using LFB staining (Supplementary Figure S1). Representative images shown here indicated that there was a marked decrease in normal-appearing myelin in vehicle treated rats as compared to that of sham operated rats. This loss of myelin was also markedly decreased in the PRM treated rats. It is important to note that at the lesion site, the normal architecture of the spinal cord was disrupted substantially, and there was a significant decrease in percentage of normal-appearing myelin in vehicle-treated animals (*p* < 0.001) which was significantly improved in PRM treated rats (*p* < 0.05). When lesion samples from PRM-treated rats and vehicle-treated rats were compared, a significant attenuation in the loss of myelin (*p* < 0.05) and better preservation of the normal spinal cord architecture was seen with the PRM treatment.

**Figure 4:**
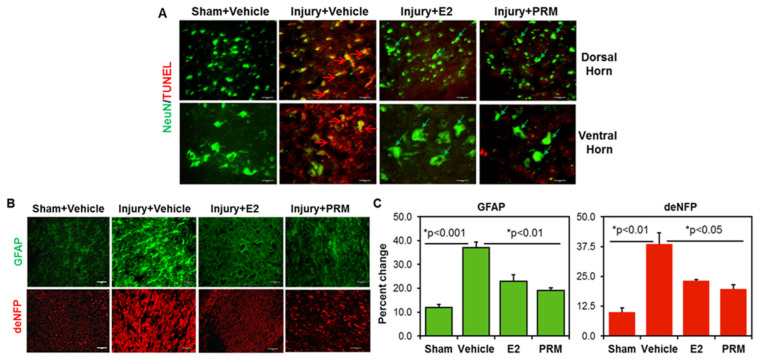
PRM decreased neuronal cell death and axon/myelin damage at day 7 following SCI. (**A**) Neural cell death was remarkably detected in vehicle treated injured rats as determined by double immunofluorescence staining using NeuN (neuron marker, green) and TUNEL (cell death marker, red). TUNEL positive cells are present in dorsal and ventral regions of injured spinal cords from vehicle treated groups as indicated by red arrows in the merged image (yellow). (Magnification, 200×, *n* = 4–6). PRM treatment markedly reversed neuron death as compared to vehicle treatment (green arrows). E2 treatment was used a positive control. (**B**,**C**) Immunohistochemical analysis of SCI tissues with astroglial marker GFAP showed significant reduction of astroglial reactivity in PRM treated injured rats (**B**, upper panel). Quantitative analysis of GFAP images by ImageJ (National Institutes of Health (NIH), Bethesda, MA, USA) showed that the effect of PRM on reducing astrogliosis was similar to that observed in E2-treated rats and was used as a positive control (**C**, left panel). Representative immunofluorescent image using an antibody against GFAP (green) is shown (Magnification, 200×, *n* = 4–6). Both PRM and E2 treatment reduced axonal damage at day 7 after SCI as indicated by a decrease in immunofluorescent staining of deNFP, a marker of axonal degeneration (**B**, lower panel; **C**, right panel). Representative immunofluorescent staining image using antibody against deNFP (red) is shown (magnification, 200×, *n* = 4–6). Data suggest that PRM promotes cell survival following SCI.

The effects of PRM on neuronal death at 7-day post-SCI were assessed by double label immunofluorescent staining using NeuN (neuron marker, green) and TUNEL (apoptosis marker, red) in both the dorsal and ventral horn regions of the spinal cord (Figure 4A). TUNEL positive cells were absent in the sham animals treated with vehicle. The injured rats treated with vehicle showed extensive TUNEL positive cells in both dorsal and ventral regions of the spinal cord (Figure 4A, red arrows). Importantly, the majority of these cells co-stained for the neuronal marker NeuN (red arrows), suggesting neuronal death in vehicle treated injured rats. In contrast, PRM treatment led to a decrease in neuronal death indicated by the low number of TUNEL positive cells and increased number of NeuN positive cells (green arrows), and the effect was similar to that observed in E2-treated rats used as positive controls. While NeuN is predominantly found in the nucleus, it is also found in the cytoplasm of neurons. In Figure 4, the expression of NeuN is shown in both nucleus and cytoplasm. This is not surprising since partially damaged neurons in the caudal injured cord could release NeuN from the nucleus into the cytosol. Even treatment with E2 or PRM may not completely protect neurons, but they may remain functional. Since the staining was performed with the TUNEL, the localization of NeuN was not clearly marked with the DAPI.

Since inflammatory events such as gliosis due to reactive glial cells are a major source of secondary damage, astrogliosis was examined in spinal cord tissue following SCI. Immunohistochemical analyses detected severe astrogliosis (GFAP) in vehicle treated SCI rats (Figure 4B, upper panel), which was significantly attenuated by PRM (Figure 3B,C). There was also a remarkable inhibition of GFAP protein expression in E2-treated rats, which served as a positive control (Figure 4B,C). Axonal damage is considered as one of the important pathological features of SCI [42] and is often associated with glial activation and inflammation. Here, axonal damage was assessed by immunofluorescent staining for dephosphorylated neurofilament (deNFP), a marker of degenerated axons. The expression of deNFP was significantly increased in the injured spinal cord at day 7 post-SCI in the vehicle treated rats as compared to sham animals (Figure 4B, lower panel). PRM treatment following injury led to significant protection from axonal damage as indicated by a decrease in the levels of deNFP and the effect was similar to E2 treatment (Figure 4B,C) at day 7 after SCI.

### 3.3. PRM Alters the Balance of Pro- and Anti-Apoptotic Proteins in Favor of Cell Survival

**Figure 5:**
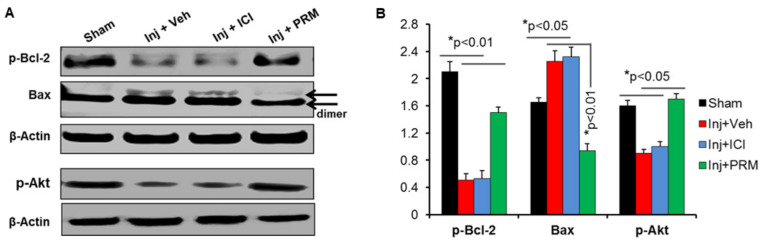
PRM treatment decreased apoptotic Bax, increased survival p-BCL-2 and upregulated p-Akt proteins at day 7 after SCI. (**A**) Western blot analysis of spinal cord samples indicates that PRM treatment for 7 days decreases the levels of pro-apoptotic Bax as well as increasing the levels of pro-survival p-BCL-2 and phospho-Akt, suggesting a protective effect. Arrows indicate a marked reduction of Bax dimer in PRM treated injured rats. (**B**) Quantitative analysis of protein bands sowed that PRM treatment significant reduced apoptotic Bax as compared to vehicle group (*n* = 6). Data suggest that PRM promotes cell survival following SCI.

To investigate whether neuronal death observed in TUNEL assay correlated with the expression of apoptotic protein in injured spinal cord, the tissue samples were analyzed by Western blotting. Decrease in the levels of the pro-apoptotic Bax protein was detected following PRM treatment in the injured spinal cord at day 7 after SCI along with a parallel increase in the levels of the anti-apoptotic protein p-BCL-2 (Figure 5A,B; *p* < 0.01). The effects of PRM in downregulating the expression of Bax dimer (arrows) and upregulating p-Bcl-2 proteins in injured rats indicate that PRM inhibits cell death and may support cell survival in SCI. It is well-known that PI3K/Akt is directly related to the survival of cells, and it plays an important role in the process of resisting apoptosis and promoting the survival of neuronal cells. Thus, the expression of p-Akt (active Akt) was analyzed by western blotting where p-Akt expression was significantly reduced in vehicle + injury and injury + ICI groups (Figure 5A,B), indicating that cell survival was reduced which was consistent with the decreased p-BCL-2 expression. The expression of p-Akt was significantly increased following treatment with PRM (*p* < 0.05), suggesting that the survival of neuronal cells in the injured spinal cord was restored by PRM. The reduction of apoptotic Bax, restoration of anti-apoptotic p-BCL-2, and upregulation of p-Akt survival signal by PRM may play crucial roles in protecting neurons after SCI.

### 3.4. PRM Attenuates Inflammatory Events following SCI

**Figure 6:**
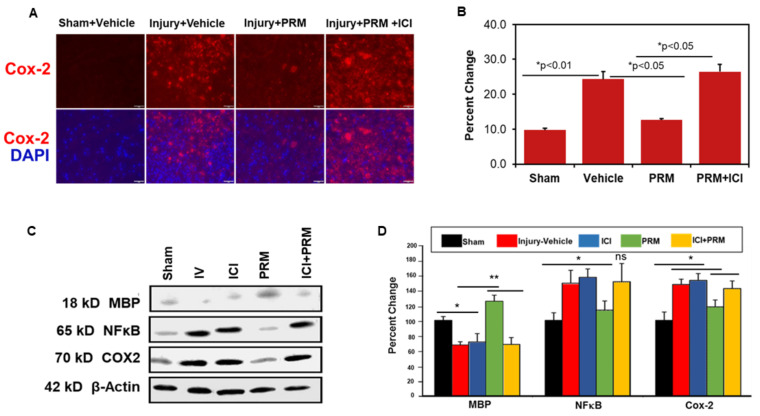
PRM decreased inflammation and reversed myelin loss at day 7 after SCI. Immunostaining indicates reduced levels of COX-2, a major inflammatory mediator following PRM treatment compared to vehicle-treated rats (**A**,**B**), and this was shown in western blot analysis as well (**C**,**D**). Representative immunofluorescent image for COX-2 (**A**) and quantitative analysis (**B**) are shown (IHC image magnification, 200×, *n* = 4–6). Western blot analysis also showed show a drop in the levels of another inflammatory mediator, NF-κB following PRM treatment compared to vehicle-treated rats (**C**,**D**). PRM was also shown to reverse myelin loss after injury indicated by an increase in MBP following PRM treatment (**C**,**D**). Representative data on western blot analysis of MBP, NF-κB, and COX-2 (**C**), and densitometry values of corresponding bands (**D**) are shown (*n* = 6–7). * *p* < 0.05, ** *p* < 0.01 vs. sham and/or injury vehicle (*n* = 6).

Following SCI, animals were treated with either vehicle or PRM, and the expression levels of several pro-inflammatory proteins were determined by immunohistochemistry and Western blotting. COX-2 immunostaining clearly indicated reduced levels of protein expression following PRM treatment compared to vehicle-treated rats (Figure 6A,B), which was confirmed by western blot analysis (Figure 6C,D). PRM treatment also attenuated the levels of another inflammatory mediator, NF-κB (Figure 6C,D), as shown by Western blot analysis. These reductions in inflammatory mediators were correlated with preservation of myelin, as indicated by increased levels of MBP following PRM treatment (Figure 6C,D; ** *p* < 0.01).

### 3.5. PRM Improves Microvessel Growth and Increases Expression of Multiple Markers of Angiogenesis

**Figure 7:**
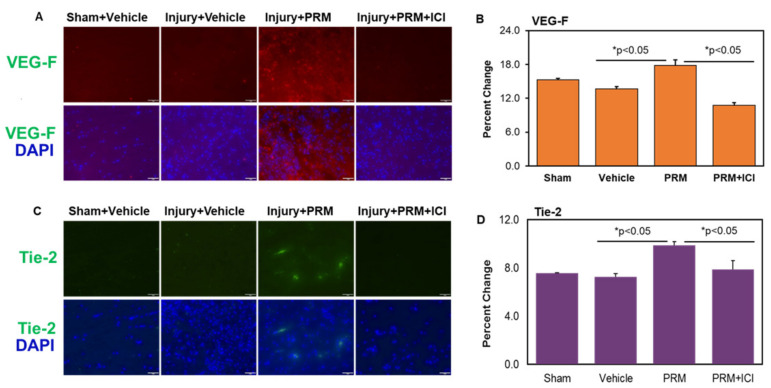
PRM improved angiogenesis at day 7 following SCI. PRM treatment increased the expression levels of angiogenic proteins VEG-F (**A**,**B**), and Tie-2 (**C**,**D**) in SCI as determined by immunohistochemistry and quantitative image analysis. Both angiogenic markers were slightly decreased in vehicle treated rats which were significantly elevated after PRM treatment. The observed increase in angiogenic protein expression by PRM injured rats was reversed by ER antagonist ICI (**B**,**D**). Representative immunofluorescent staining images using antibodies against VEG-F and Tie-2 are also shown (**A**,**C**) along with corresponding quantitation (**B**,**D**) (magnification, 200×, *n* = 4–6).

An increase in the expression of major angiogenic markers VEG-F (Figure 7A,B) and Tie-2 (Figure 7C,D), as determined by immunofluorescent staining and corresponding densitometric analyses, indicated PRM treatment improved angiogenesis in the subacute phase (day 7) of SCI. In the presence of the ICI inhibitor, the levels of VEGF, a major growth factor promoting blood vessel growth (red), and Tie-2, an important angiopoietin receptor (green), were brought to the levels of the control groups (Figure 7).

**Figure 8:**
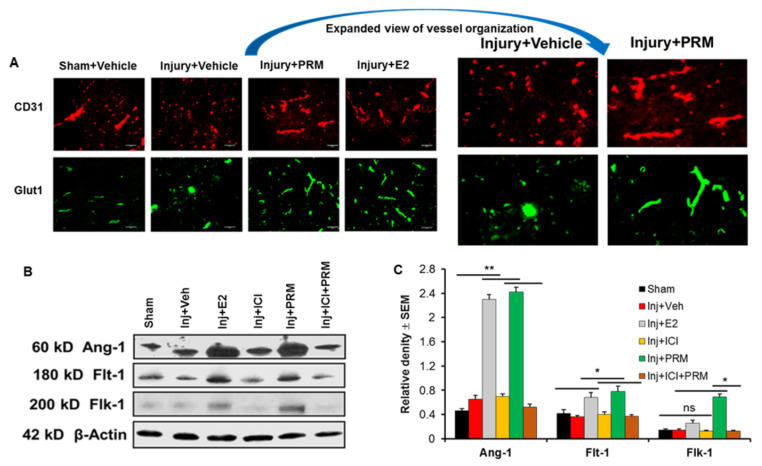
PRM treatment for 7 days increased the expression of CD31 and GLUT1 angiogenic markers in SCI. (**A**) PRM improved angiogenesis in penumbra at day 7 post-SCI indicated by an increase in immunostaining of the angiogenesis markers CD31 and GLUT1. Expanded view of CD31 and GLUT1 in injury + vehicle vs. injury + PRM represents marked differences in vessel organization. The effect of PRM on increased angiogenesis was similar to that observed in E2-treated rats (positive control) following SCI. Representative immunofluorescent staining images using antibody against CD31 (red) and GLUT1 (green) are shown (magnification, 200×, *n* = 4–6). (**B**) PRM treatment also increased the expression of other angiogenesis-related proteins, such as Ang-1, Flt-1, and Flk-1, as shown by representative western blotting. (**C**) E2 and PRM significantly increased angiogenic markers in injured spinal cord as determined by densitometric analysis using ImageJ software, NIH, Bethesda, MA, USA). * *p* < 0.05, ** *p* < 0.01. ns = not significant.

The expression levels of other markers for microvessel growth and angiogenesis were also examined by immunohistochemistry and Western blotting at day 7 after SCI. Microvessel growth was examined using CD-31 and GLUT-1 antibodies as markers of microcapillaries in the injured rats and found to be substantially reduced compared to sham (Figure 8A). CD-31 (red) and GLUT-1 (green) staining indicated a loss of normal vessel organization in the penumbra of SCI compared to sham control. Examination of the effects of PRM at 7-day post-injury showed markedly increased microvessel growth compared to injury/vehicle treatment as indicated by increased expression of CD31 and GLUT-1 (Figure 8A). E2 treatment worked similarly to PRM to restore the vasculature.

PRM treatment also significantly increased the expression of a number of other angiogenesis markers, including Ang-1 and the VEGF receptors Flt-1 and Flk-1 (Figure 8B,C). Similar increase in the expression of these proteins were also found with E2 treatment. Increased expression of these proteins following PRM treatment was also significantly reduced by treatment with the ER antagonist ICI (10 µM) (Figure 8B,C), indicating possible involvement of ER-mediated pathways in angiogenesis. These data suggest that PRM treatment can substantially increase microvessel growth and improve angiogenesis in the subacute phase (at day 7) of SCI.

### 3.6. PRM Increased Expression of Erα and Erβ but Not GPR30

**Figure 9:**
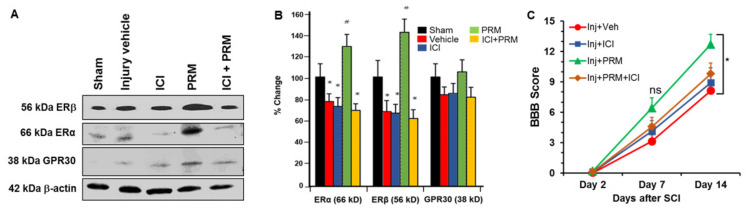
PRM treatment for 7 days increased expression of E2 receptors following SCI and promoted improved motor function in SCI. PRM treatment increased the expression of E2 Receptors, Erα and Erβ, following SCI as shown by Western blotting (**A**), and densitometric analysis of protein bands (**B**). In contrast, PRM did not significantly alter the levels of GPR30, a different E2 receptor (**A**,**B**), suggesting the increased expression of Erα and Erβ receptors is generated by PRM treatment. The increase in the levels of ER receptors was reversed by the addition of ER antagonist (ICI), further indicating that the neuroprotective action of PRM is based on their specificity to both E2 receptors. *n* = 10. * *p* < 0.05 compared to sham animals, and # *p* < 0.05 compared to vehicle treated SCI animals. (**C**) BBB score was determined for SCI rats treated with vehicle control, ICI control, PRM, and PRM + ICI as described in the methods. Rats were graded weekly using the BBB scale to examine motor function after SCI. PRM treated rats showed significantly higher BBB scores compared to controls or ER inhibitor (ICI) at day 14. * *p* < 0.05, ns = not significant.

PRM treatment caused a significant increase in expression of Erα and Erβ at day 7 after SCI (Figure 9A,B). However, a similar rise was not observed for GPR30 receptor expression. Co-treatment of PRM with ICI (ER antagonist) significantly inhibited Erα and Erβ expression, indicating the neuroprotective effects of PRM might be receptor mediated.

### 3.7. PRM Improves Motor Function in SCI Rats

While PRM treatment decreased glial activation and inflammation, and induced neuroprotection in SCI, the functional outcome especially the status of motor function remains unknown. Thus, rats treated with vehicle, ICI, PRM, and a combination of PRM + ICI were followed for 2 weeks post-injury and BBB locomotor scores were recorded weekly until sacrifice of the rats. BBB locomotor scores recorded in appropriate controls were compared with the PRM therapy showing a significant improvement in functional outcome in PRM treated rats at day 14 (Figure 9C). While all other parameters in the PRM treated rats were analyzed at day 7, BBB locomotor scores were monitored on day 2, day 7, and day 14 because the differences in functional outcomes between control and treatment groups were commonly detected after day 14. Data obtained from these studies suggested that PRM treatment significantly improved motor function in SCI rats at day 14 as compared to vehicle treated controls (*p* < 0.05).

### 3.8. Major Side Effect of PRM Treatment

While PRM treatment provided neuroprotection and some functional improvement, half of the animals treated with PRM exhibited a major urogenital tract issue. Thick, dark blood was produced in the urine at approximately at day 5 post-injury. This was accompanied by pale eyes and lethargic behavior. However, this issue was resolved after stopping the PRM treatment at 7 days, and the functional behavior was improved. Interestingly, some animals had no bladder issues (36%) while others followed a normal timeline for bladder function (14%).

## 4. Discussion

Tissue damage in SCI is multi-factorial and requires a therapeutic approach that can attenuate a majority of the destructive pathways and promote recovery of motor function. While promising therapeutic agents including melatonin, have shown potential for ameliorating the damage and providing neuroprotection to the injured spinal cord [17,18,19], none have been found more beneficial than treatment with E2 [14,15,16]. E2 at a very high non-physiological dose (4 mg/kg) administered intravenously to rats with SCI has been found to provide neuroprotection and improve function [7,11,12]. However, the potential side effects prevent use of such high doses of E2 in a clinical setting. For example, the systemic administration of high dose E2 will most likely induce deep vein thrombosis, a common detrimental side effect in human SCI. Therefore, much lower doses of E2 (1–10 μg/kg) have been found to be neuroprotective with decreased inflammation, increased angiogenesis, and improved locomotor function [14,15,16]. During our investigation on the effects of PRM on SCI, E2 remained unapproved by the FDA for use in the clinic. However, PRM is now FDA-approved for clinical applications. Therefore, in the current investigation, comparable doses of the conjugated estrogens PRM (100 μg/kg PRM), which is equal to approximately 8–10 μg of E2), likewise attenuated cell death and inflammation and promoted angiogenesis in SCI. These studies also demonstrated that the effects of PRM are primarily due to E2 and not due to the other sterols present in PRM. This is confirmed by using the pan-ER inhibitor ICI182780 since the beneficial effects of E2 are mediated by ERα and ERβ.

Tissue damage following SCI involves inflammatory pathways, exacerbated by interruptions of normal blood flow which leads to cell death, disruption of the axon–myelin structural unit, and loss of function. PRM at a low dose range (100 μg/kg ≈ 10 μg/kg E2) significantly decreased inflammatory factors NF-κB and COX-2 (Figure 6) and attenuated neuron death at day 7 in post-SCI. A similar protection of cells and reduction of inflammation in SCI by a much higher dose range of PRM (1 mg/kg ≈ 100 μg/kg E2) as well as melatonin, a potent antioxidant scavenging both reactive oxygen species (ROS) and reactive nitrogen species (RNS) in the cells, has been recently demonstrated [17,19,22,33,43,44]. However, the results presented in the current report with low dose of PRM seem to have more clinical relevance compared to the previous studies. Nonetheless, E2 the main active component in the PRM formulation, has been shown to be neuroprotective following traumatic brain injury (TBI) [45,46,47,48]. Moreover, its role in modulating Ca^2+^ influx following neuronal damage was demonstrated in an in vitro model of neuronal injury [49]. Similar E2-mediated beneficial effects have been reported in focal and global ischemia as well as in experimental autoimmune encephalomyelitis (EAE), which is a widely used animal model of multiple sclerosis (MS) [50,51,52,53,54,55].

Treatment of SCI with PRM, such as E2 and melatonin, provides similar protection by reducing astrocytic reactivity and pro-apoptotic Bax, preventing axon and myelin degeneration and neuron death by increasing anti-apoptotic pAkt [14,18,22]. These parameters are inhibited by the ER antagonist ICI182780, again indicating PRM efficacy in SCI, such as E2, involves E2 receptors. Disruption to blood vessels occurs immediately following SCI, leading to ischemic damage to cells. Therefore, use of beneficial agents such as E2, melatonin, and VEGF that are capable of promoting microvessel growth and improving restoration of blood supply to the damaged area for cell survival is imperative [17,56,57]. Otherwise, cell death and neurodegeneration occur, leading to loss of function. E2 has been linked to promotion of angiogenesis following SCI [14]. Its angiogenic involvement in the female reproductive neovascularization is mediated by increased vascular endothelial growth factor (VEGF), ERα, and basic fibroblast growth factor (bFGF) expression [58,59]. Both VEGF and bFGF are increased following SCI [60,61,62,63]. Angiogenic proteins Angiopoietins (Ang-1 and Ang-2) and Tie-1/Tie-2 receptors are present in endothelial cells and are elevated in SCI following treatment with E2 [14]. PRM treatment, like E2, demonstrated increased expression of angiogenic factors, Tie-2, Ang-1, and microvessel growth promoters, such as CD31 and GLUT-1. Thus, the improved blood supply rendered by PRM treatment may contribute to cell protection and functional recovery. Such upregulation of endothelial cell proliferation and microvessel growth has been shown in cerebral ischemia and SCI [14,33,64,65]. Angiogenesis and neuroprotection rendered by E2, melatonin, and VEGF are known to be mediated by their respective receptors [7,11,17,22,60].

E2 efficacy is known to be mediated primarily by its two receptors Erα and Erβ, which are present in neurons and glia [66,67,68,69]. E2 promotes neuronal survival and neurite growth and attenuates neurodegeneration in ischemia, Alzheimer’s disease (AD), EAE, and SCI [16,50,51,70,71,72]. These neuroprotective effects of E2 have been attributed to upregulation of Erα and Erβ. To demonstrate that the effects of PRM are primarily due to E2 receptors Erα and Erβ and not a result of progestin or other sterols in the conjugate, experiments have been designed using PRM in the absence and presence of ER antagonist ICI182780 in SCI rats. As shown previously, E2 treatment of SCI upregulated Erα and Erβ, attenuated inflammation, protected cells, and improved locomotor function. Likewise, PRM treatment in the absence of ER antagonist ICI182780 attenuated inflammation, protected cells, and upregulated Erα and Erβ compared to injury vehicle (no treatment with PRM or ICI). In contrast, PRM treatment in the presence of antagonist ICI abolished all beneficial effects rendered by PRM alone. This suggests that the PRM effects are mediated through Erα and Erβ and not by progestin or other components present in the conjugated PRM. Therefore, PRM (like E2) may be taken into the clinic for its translational potential. PRM (100 µg) use in the clinic (following IND approval), has been found to be safe without any significant detrimental effects (unpublished, Varma et al.). PRM treatment is known to replace E2 during menopause, strengthen bones, relieve symptoms of menopause and help with the pain. However, PRM can increase the risk of cancer of the breast, uterus, or ovaries and unusual vaginal bleeding in women if used for a long term. Depending on the dose, an increased concentration of PRM can also elevate the risk of blood clot and liver disease in certain populations. These side effects can be minimized by reducing the dose of PRM.

In summary, the data on various parameters indicate that PRM treatment at 100 μg/kg (equivalent to approximately 10 μg/kg E2) reduced inflammatory factors NF-κB and COX-2, decreased activity of astrocytes and microglia, upregulated cell survival proteins, protected neurons, and preserved myelin and axons following SCI. Most importantly, PRM promoted microvessel growth by upregulating angiogenic factors and E2 receptors, which are thought to mediate the beneficial effects of both PRM and E2. Co-treatment of acute SCI animals with PRM and the ER antagonist ICI182780 reversed the beneficial effects, further indicating the involvement of ERs. Although the reduction of inflammatory response, protection of neurons, and preservation of axon and myelin by PRM were comparable to E2 at day 7, the recovery of locomotor function in SCI rats was not significantly improved by PRM at that time point. However, our previous study with E2 treatment showed a significant recovery of locomotor function at day 7, and this improvement continued up to 4 weeks following SCI [15]. In contrast, PRM treatment improved functional recovery in SCI rats and showed a strong trend in recovery of locomotor function at day 7, suggesting that the efficacy of E2 may be superior to PRM in functional outcome. This finding further confirms the novelty of E2 as a powerful agent for therapy of SCI. Whether this difference in motor function is due to the effects of other steroids in the PRM conjugates or the major side effects detected (dark thick blood in the urine) is not clear. However, our study suggests that there are many beneficial effects of PRM in SCI although few side effects also were noted. This is consistent with other studies in animals as well as in humans where use of E2 conjugates showed a number of beneficial effects with some side effects including unusual vaginal bleeding in menopausal women [73,74,75]. Overall, the PRM actions were almost similar to E2 effects in SCI, indicating that PRM can be used as an effective therapeutic agent to improve post-SCI outcomes.

## Data Availability

Not applicable.

## Supplementary Materials

The following supporting information can be downloaded at: https://www.mdpi.com/article/10.3390/ijms23042384/s1.
