# Supplementary material for: Premarin Reduces Neurodegeneration and Promotes Improvement of Function in an Animal Model of Spinal Cord Injury
Source: Int J Mol Sci. 2022 Feb 21;23(4):2384. doi: 10.3390/ijms23042384 (PMC8875481; doi:10.3390/ijms23042384)
Supplement: Supplementary file 1 [file ijms-23-02384-s001.zip › ijms-1574923-supplementary.pdf]

International Journal of
Molecular Sciences

MDPI

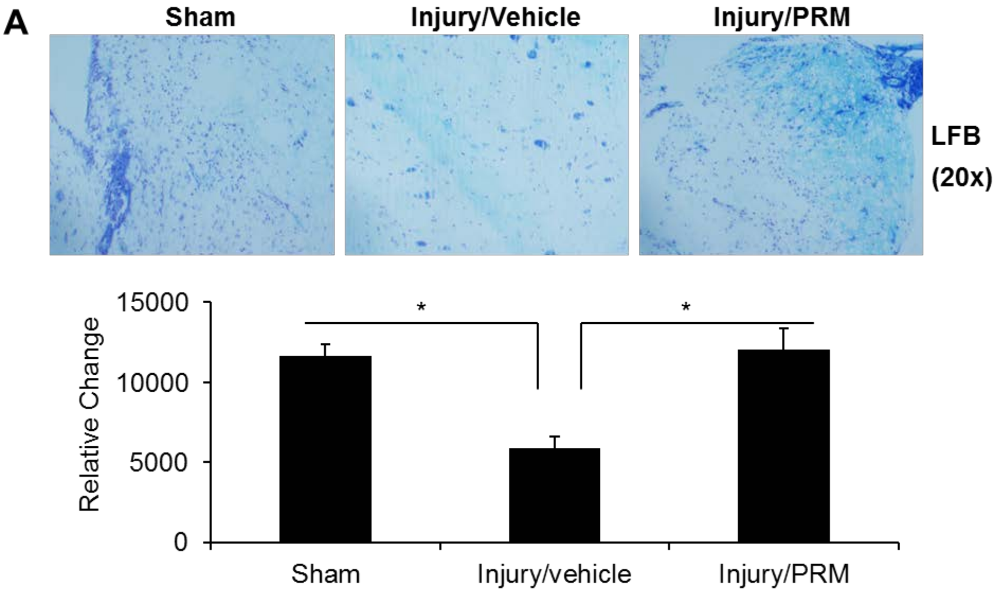

Figure. S1. Thin (10 μm) longitudinal spinal cord lesion sections were stained with LFB as described in the methods. Representative samples from sham, injury/vehicle, and injury/PRM were assessed for myelin integrity. (A) Microscopic images from the lesion segment show demyelination, which was partly restored following PRM treatment. (B) Image analysis was performed by ImageJ. Significant difference between vehicle- and PRM-treated rats was indicated by $p<0.05$ (n=4-5). Significant difference from sham and vehicle-treated rats was also indicated by $^*p<0.05$. Magnification, 20X.
